# The Position of Women at Cambridge

**Published:** 1920-11-06

**Authors:** 


### THE EDITOR'S LETTER-BOX.

**Correspondence on all subjects is invited, but we cannot in any way be responsible for the opinions expressed by our correspondents, who must give their name and address as a guarantee of good faith, but not necessarily for publication. Correspondents are reminded that brevity of style and conciseness of statement greatly facilitate early insertion.**

### The Position of Women at Cambridge.

### SOME UNIVERSITY OPINIONS.

*To the Editor of* The Hospital.

Sir,—It is far from certain that the admission of women to membership of the University would be by any means the best for Cambridge as an educational centre—either for the men or the women. The time spent at Cambridge should be spent in work to fit a man for the struggle in life, to meet other men and learn their views and to mature his own. Would this end be kept so well in view in the presence of women? Surely not. Even now there is far too much of the sex influence among undergraduates; chaperonage has become largely a thing of the past; dances for men and women students are held two or three times a week; it has been found that in laboratories the demonstrators are apt to be more assiduous in helping the women than the men.

All this must be most disturbing to steady work in the case of the men, and the same applies to the women's side of the question. Some women, no doubt, are capable of working amongst men quite undisturbed, but the majority would find their concentration at fault. Should women be admitted, many more would pass to their present colleges, and doubtless other women's colleges would arise. Oxford has already experienced this increase in the numbers of women. Would it not be well to keep one university in the United Kingdom for men only, where study could be pursued unhampered by the insidious influence of the opposite sex?

It was the late Professor Sir William Osler who said of the man who took up medicine, that he must put his heart on ice if he wished to do good work; and the same wise advice applies to all branches of study.

Pass now to the argument that Oxford has admitted women; it has been admitted by one of the supporters at Oxford that cowardice was at the bottom of the vote; indeed, one of the supporters wrote in *The Times* of May 25 an eloquent plea for a women's university.

In America, of three independent (not State-aided) universities—Princeton, Yale and Harvard—the first has no women students. Yale has no undergraduate women, but admits to post-graduate studies. Harvard has Harvard conferring degrees on women, and Radcliffe College conferring degrees on women, but men are not lectured together, apparently.

Is it possible that it is degrees only that women are working for? Some women would be content to have the privilege of placing B.A. or M.A. after their names, but it is difficult to see how this could be allowed without conferring on them all the privileges of the degree. It is more probable that power in the university is what the majority are aiming at.

Cambridge will do best to maintain a masculine independence and remain a centre for the highest education of men—the only one left in the United Kingdom.—Yours faithfully, A Member of the Senate.

